# Practical Guidelines for Diagnosing and Treating Thyroid Disease Based on the WOMED Metabolic Model of Disease Focusing on Glycolysis and Coenzyme Q_10_ Deficiency—A Clinical Alternative to the 2021 Retired Clinical Practice Guidelines of the Endocrine Society

**DOI:** 10.3390/diagnostics12010107

**Published:** 2022-01-04

**Authors:** Roy Moncayo, Helga Moncayo

**Affiliations:** WOMED, Karl-Kapferer-Strasse 5, 6020 Innsbruck, Austria; anmeldung@womed.at

**Keywords:** thyroid, magnesium, selenium, coenzyme Q_10_, ferritin, mitochondria, oxidative phosphorylation, glycolysis, hypoxia

## Abstract

This review aims to provide a functional, metabolic view of the pathogenesis of benign thyroid disease. Here, we summarize the features of our previous publications on the “WOMED model of benign thyroid disease”. As of 2021, the current state of art indicates that the basic alteration in benign thyroid disease is a metabolic switch to glycolysis, which can be recognized using 3D-power Doppler ultrasound. A specific perfusion pattern showing enlarged vessels can be found using this technology. This switch originates from an altered function of Complex I due to acquired coenzyme Q_10_ deficiency, which leads to a glycolytic state of metabolism together with increased angiogenesis. Implementing a combined supplementation strategy that includes magnesium, selenium, and CoQ_10_, the morphological and perfusion changes of the thyroid can be reverted, i.e., the metabolic state returns to oxidative phosphorylation. Normalization of iron levels when ferritin is lower than 50 ng/mL is also imperative. We propose that a modern investigation of probable thyroid disease requires the use of 3D-power Doppler sonography to recognize the true metabolic situation of the gland. Blood levels of magnesium, selenium, CoQ_10_, and ferritin should be monitored. Thyroid function tests are complementary so that hypo- or hyperthyroidism can be recognized. Single TSH determinations do not reflect the glycolytic state.

## 1. Preamble

The practice of good clinical medicine practice requires the exact recognition of fundamental biochemical processes that cause disease. Acquiring this knowledge will allow practitioners to provide an adequate treatment that will modify the key processes of pathogenesis. This goal setting corresponds to concepts brought by Stetenga looking at medical interventions and their effectiveness [1]. A key concept from this publication states the following: “To be effective, a medical intervention must improve one’s health by targeting a disease”. This goal coincides with the principles of effectiveness and efficiency pointed out by Archie Cochrane [2].

**Figure 1:**
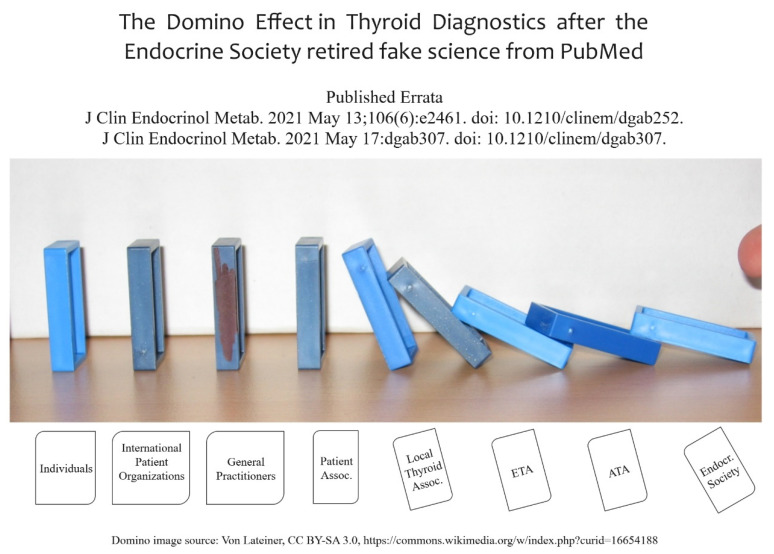
Domino effect of retiring clinical practice guidelines affecting thyroid societies and individuals.

Following the principles related to evidence of research findings and clinical practice [3], a series of guidelines for clinical practice commenced appearing in the 1990s [4]. The Institute of Medicine Committee included the following statement on validity: “VALIDITY: Practice guidelines are valid if, when followed, they lead to the health and cost outcomes projected for them” (p. 10, [4]). The field of clinical practice and thyroid diseases has been shattered in 2021 by an unprecedented action taken by the Endocrine Society. On 26 February 2021, i.e., fourteen years after the publication by Abalovich, Amino, Barbour, Cobin, De Groot, Glinoer, Mandel, and Stagnaro-Green (PMID 17948378), the Journal of Clinical Endocrinology & Metabolism has retired the guideline [5]. The same happened to the 2012 update of the guideline [6] which was written by De Groot, Abalovich, Alexander, Amino, Barbour, Cobin, Eastman, Lazarus, Luton, Mandel, Mestman, Rovet, and Sullivan (PMID 22869843). This action will certainly have a large impact on medical practice since many publications were built upon these misconceptions (Figure 1). It is quite unfortunate that the Endocrine Society did not give precise information about this critical procedure. We have chosen to use the PMID identifiers to avoid any unnecessary citation of these discredited articles.

“Science is built of facts the way a house is built of bricks: but an accumulation of facts is no more science than a pile of bricks is a house.”

Henri Poincaré (1854–1912)

Lateiner, CC BY-SA 3.0, https://commons.wikimedia.org/w/index.php?curid=16654188 (accessed on 18 December 2021).

In the following sections, we will summarize our biochemical function-related model of acquired thyroid disease, which aims at restoration of the thyroid, thus restoring health.

## 2. Introduction

This publication aims to demonstrate concisely our understanding of the metabolic pathogenesis of thyroid disease and to describe the biochemical interventions needed for a successful treatment. Within the context of evidence in medicine, Chambless and Hollon presented the following statement: “…efficacy must be demonstrated in controlled research in which it is reasonable to conclude that benefits observed are due to the effects of the treatment and not to chance or confounding factors…” [7]. Following this principle, we will emphasize the importance of 3D-power Doppler ultrasound examination of the thyroid for the evaluation of the morphology and perfusion of the gland. This methodology has allowed us to recognize that increased thyroid perfusion can be seen in hyperthyroidism, hypothyroidism, during pregnancy, postpartum, or post-COVID-19 infections. The main biochemical correlate of this situation is coenzyme Q_10_ (CoQ_10_) deficiency. Based on recent experimental studies by Liparulo et al. concerning the effect of CoQ_10_ synthesis inhibition on the metabolic switch to glycolysis [8], we can now interpret our sonography findings as being indicative of glycolysis—and hypoxia—in the thyroid. We have been able to confirm this hypothesis through the use of diagnostic imaging using ^18^F-fluorodeoxyglucose positron emission tomography (Figure 6 in [9]).

Besides CoQ_10_ deficiency, thyroid disease patients are also deficient in magnesium, selenium, and iron. When these deficiencies are corrected by supplementing the missing elements, we have documented reconstitution of thyroid morphology and perfusion [10]. By this, our approach coincides with the requirements for measuring effectiveness advanced by Stetenga asking for the use of a good measuring instrument together with an adequate measure of outcome [11].

Ultrasound and 3D-perfusion evaluations of the thyroid have never been included in clinical practice guidelines coming from expert consensus meetings. The same applies to laboratory determinations of magnesium, selenium, CoQ_10_, and iron. These conceptual limitations do not allow us to compare our results with the medical literature dedicated to thyroid diseases.

In the following sections, we will proceed to delineate our clinical practice procedure for patients with thyroid disease.

## 3. The Clinical Approach

Over two and a half decades, we have conducted consecutive observational studies, sequentially adding elements to the concepts of an acquired mitochondrial disorder and altered function of the endoplasmic reticulum referring to protein repair. Our clinical work has also dealt with the topics of thyroid function and female fertility [9,10,12,13,14,15,16,17,18,19,20,21,22,23,24,25,26,27,28]. Every patient referred to an examination of the thyroid should go through detailed clinical history taking and ultrasound examination that includes 3D-power Doppler evaluation (Table 1).

## 4. Interpretation of Thyroid Ultrasound and 3D-Power Doppler Perfusion

The classical description of chronic thyroid disease was made by Hashimoto in 1912 [29]. In Table 2, we compare the findings of Hashimoto with the relevant biochemical and sonography parameters involved in thyroid disease. The original text in German used by Hashimoto is included in the heading of the corresponding columns.

The findings of the sonography examinations can be translated into a descriptive table indicating which therapeutical approach is needed (Table 3).

Our clinical practice has brought us to the following prescription recommendations. Magnesium deficiency should be treated with pure magnesium citrate prepared as a magistral formulation at a dose of 3.5 to 4.0 g per day dissolved in water and taken during the day. The amount of elemental magnesium in the magnesium citrate salt corresponds to approximately 8% or 280 to 320 mg/day. The main theoretical advantage of this formulation lies in the ability of citrate to localize inflammation [30]. For the correction of selenium and CoQ_10_ deficiencies, we have relied on products from Pure Encapsulations^®^ for 15 years and have obtained satisfactory results. The preparations used include selenomethionine (200 µg capsule) and CoQ_10_ (60 mg capsule). Supplementation begins with 1 capsule of selenomethionine and CoQ_10_ daily taken together at night during the first 2 weeks. Beginning on the 3rd week, the dose is reduced to only 3 capsules per week of each preparation taken on alternate days. Treatment costs amount to €220 per year or €0.66 per day. The efficacy of supplementation is controlled by laboratory determinations of these parameters. The target values to be reached through supplementation are >0.9 mmol/L for magnesium, >80 µg/dL for selenium, and >1200 µg/L for CoQ_10_. Ferritin levels should be >50 ng/mL. Power Doppler sonography examinations deliver in vivo information as to the levels of these nutrients and can also be used to monitor the supplementation therapy [10].

**Figure 2:**
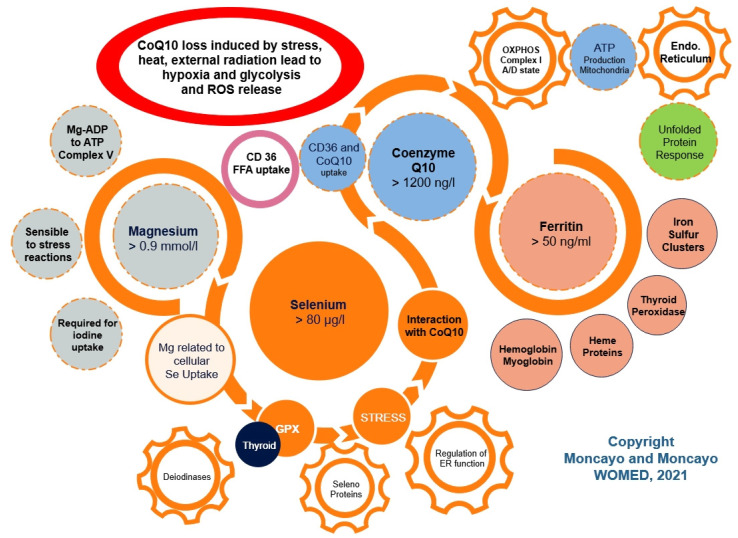
A simple model of mitochondrial complexity showing interactions of magnesium, selenium, CoQ_10_, and iron in relation to the function of mitochondria and the endoplasmic reticulum. The red oval depicts the critical situation of CoQ_10_ deficiency leading to glycolysis and ROS release.

The essence of our concerning approach can be taken from the abstract of the publication by Kitano on Systems biology: “To understand biology at the system level, we must examine the structure and dynamics of cellular and organismal function, rather than the characteristics of isolated parts of a cell or organism” [31]. This biological point of view has been often forgotten by researchers who have evaluated magnesium, selenium, CoQ_10_, or iron as single, isolated parts of a complex organism in relation to thyroid or heart disease. Our simple model of mitochondrial complexity is shown in Figure 2. Mitochondrial integrity is essential for the prevention of reactive oxidative reactions.

**Figure 3:**
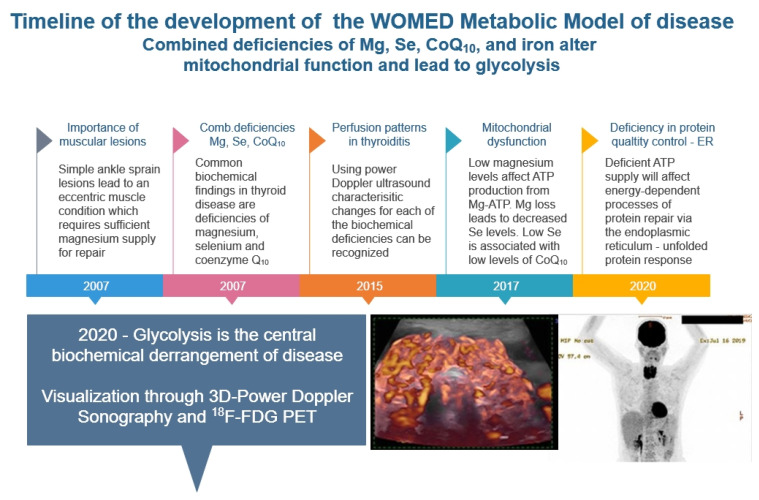
Timeline of our clinical investigations leading to the recognition of glycolysis as a key mechanism of disease affecting the thyroid and the heart.

A timeline representation of our research leading to the detection of glycolysis—as a simile of hypoxia—is shown in Figure 3. The ^18^F-FDG PET image shows the concomitant glycolytic condition of the thyroid and heart.

## 5. Discussion

Several versions of “conventional” treatment approaches for thyroid disease have appeared in the past decades [32]. The predominant thesis of treatment adequacy contained in such guidelines has been to monitor TSH by laboratory determinations [33]. One gains the impression that thyroid treatment is exclusively oriented toward achieving specific TSH levels [34]. This practice philosophy ignores the investigation of basic pathogenetic processes present in the thyroid gland as well as clinical aspects and symptoms reported by patients. It is therefore not surprising to find several reports of treated patients who complain about residual symptoms (Table 4). This situation of dissatisfaction was the starting point for our studies in 2007.

Our first study on nutrients and thyroid disease was focused on selenium, zinc, and vitamin C [19]. The identification of selenium deficiency as a common finding in benign and malignant diseases brought us back to look at the literature on this element. The starting point for investigations on the role of selenium in liver disease was set in 1957 by Schwarz and Foltz [57]. They described the beneficial effect of selenium in the context of dietary necrotic liver degeneration. In a fraction originally called Factor 3, they discovered that selenium was present. Two central observations on the relation between selenium and CoQ_10_ were done by Green et al. in 1961 [58] and by Hidiroglou in 1967 [59]. Administering selenium improved the tissue levels of CoQ_10_ in the experimental animals. A similar observation about this relation was published by Vadhanavikit and Ganther in 1993 [60]. This physiological aspect has not been considered in many studies that have chosen to study the effects of selenium or CoQ_10_ given as single agents to overcome putative oxidative changes. Another study speculated that the lowering of CoQ_10_ levels depended on the depressed GSH-Px activity that resulted from selenium deficiency [61].

In situations of selenium deficiency, mitochondrial structure and also the electron transport function were described as being altered [62]. A study on the distribution of selenium in mitochondria revealed that the highest concentration was found in the inter-membrane space, the inner membrane, and the matrix [63]. These studies delivered biochemical function concepts for our concept of acquired mitochondrial dysfunction.

The identification of glycolysis in the thyroid and the heart using diagnostic imaging methods [9] directed our attention to studies using similar methodology. The literature contains several descriptions of studies that have looked at diffuse thyroid uptake of ^18^F-FDG in PET imaging. Albano et al. recently reviewed this topic concerning thyroiditis [64]. Albano and many other authors found in the literature consider that the nature behind tracer uptake in the thyroid has remained unexplained. Although the professional background of the authors is nuclear medicine, their conclusions did not convey the real interpretation of images done with ^18^F-FDG PET, i.e., glycolysis. It is also noteworthy that an intense tracer uptake in the heart (Figure 2 in [64]) was left uncommented. Based on an ongoing literature evaluation being done by us, we believe that cardiac uptake also represents a situation of CoQ_10_ deficiency.

## 6. Conclusions

We consider that our model corresponds to epidemiological definitions when health problems are considered: “the study of the distribution and determinants of health-related states or events in specific populations and the application of this study to control of health problems” [65]. TSH alone is not the central health problem and should not be taken as the ultimate minimalistic evaluation parameter of thyroid disease. We firmly believe that diagnostic imaging with advanced sonography techniques [9,20] for the investigation of thyroid disease has been a game-changer element that has broadened our view about the real disease pathogenesis. We propose that the examination procedure described by us in this manuscript can lead to a substantial improvement of clinical practice at times when multi-author practice recommendations have been dismantled [5,6].

## Figures and Tables

**Table 1:** The clinical approach to cases where thyroid dysfunction is suspected.

Parameter	Remarks
Clinical history	Has thyroid disease been suspected before? What were the symptoms?
	Was the previous diagnosis done using “fake” reference values for TSH?
	How was the diagnostic approach? Are there any previous ultrasound examinations? What did the power Doppler pattern of thyroid perfusion show?
	Are the initial symptoms still present?
	Are there any residual symptoms? Fatigue?
Thyroid morphology	Normal structure
	Inhomogeneous pattern with some areas presenting decreased echogenicity
	Pronounced inhomogeneous pattern
	Loss of echogenicity, i.e., thyroid pattern is the same as that of muscle
	Pronounced changes with fibrosis
Thyroid perfusion	Normal
	Slight increase with a point-like pattern—suggests magnesium deficiency
	Moderate increase with a wire-like pattern—suggests CoQ_10_ deficiency
	Very intense hyper-perfusion—suggests a severe combined deficiency condition
Basic laboratory parameters	Magnesium, ferritin
	CK and NT-proBNP
	Selenium and CoQ_10_
Thyroid function parameters	fT3, fT4, TSH. Thyroid antibodies.

**Table 2:** Summary of the WOMED clinical approach to thyroiditis concerning the 3 main findings described by Hashimoto (in the original German description). The clinical validity of diagnostic procedures including power Doppler sonography is explained for every stage.

	“Eigenthümliche Art von Chronischer Entzündung”	“Wucherung des Gefässendothels”	“Fällt Selbst dem Schwunde Anheim”
	Chronic Inflammation	Enlarged Endothel	Destruction
TSH	no	no	Elevated in hypothyroidism
Thyroid-Abs	Partial correlation	no	no
Ferritin	no	no	no
Magnesium	yes	yes	Partly
Coenzyme Q_10_	yes	yes—relates to the dynamics of perfusion	No
Selenium	yes	no	Yes in fibrosis
Sonography	yes	yes	yes
Power Doppler Images	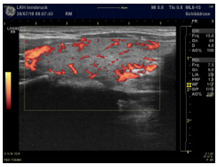	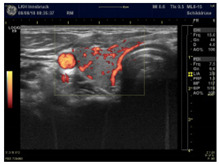	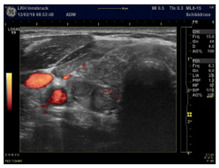
Condition	Magnesium def. pattern	CoQ_10_ def. pattern	Chronic fibrosis

**Table 3:** Interpretation matrix of 3D-power Doppler Thyroid Sonography.

Morphology	Perfusion	Magnesium	Coenzyme Q_10_	Selenium
Normal	normal			
Hypo echogenic homogeneous	fine granular	deficiency		
Hypo echogenic inhomogeneous	thickened vessels	deficiency	deficiency	
Hypo echogenic inhomogeneous with fibrosis signs	moderately increased	probable	probable	deficiency
Very low echogenic	increased	deficiency	deficiency	deficiency
Very low echogenic with fibrosis signs	not increased = final stage	-	-	-

**Table 4:** Selected citations on failed thyroid hormone replacement therapy.

Taylor (1970) [35]	“It may be the experience of many clinicians, as it has been ours, that a very small group of patients with hypothyroidism are not entirely well on thyroxine replacement alone.”
Walsh J.P. (2002) [36]	“… higher prevalence of symptoms consistent with hypothyroidism, such as impaired memory and clarity of thought, tiredness, weight gain, somatic pain and physical clumsiness.”
Saravanan (2002) [37]	“This community-based study is the first evidence to indicate that patients on thyroxine replacement even with a normal TSH display significant impairment in psychological well-being”
Weetman A.P. (2006) [38]	“The majority of patients who demand thyroid hormone treatment for multiple symptoms, despite normal thyroid function tests, have functional somatoform disorders.”
Okosieme (2016) [39]	“However, the management of patients with a sub-optimal clinical response remains challenging.”
McAnich and Bianco (2016) [40]	“The euthyroid yet symptomatic patient.”
Sheehan (2016) [41]	“Increasingly, when a physician informs a patient that their thyroid is not the cause of their symptoms, the patient is dissatisfied and even angry.”
Chaker (2017) [42]	“However, a substantial proportion of patients who reach biochemical treatment targets have persistent complaints.”
Stott (2017) [43]	“Levothyroxine provided no apparent benefits in older persons with subclinical hypothyroidism.”
Jonklaas (2017) [44]	“However, despite the successes in treating hypothyroidism, there are clearly diseases aspects of treating hypothyroidism that are not yet understood.”
Feller (2018) [45]	“These findings do not support the routine use of thyroid hormone therapy in adults with subclinical hypothyroidism.”
Yamamoto (2018) [46]	“… no evidence of benefit of levothyroxine therapy on obstetrical, neonatal, childhood IQ or neurodevelopmental outcomes.”
Mayor (2018) [47]—editorial comments on Feller	“The results, reported in JAMA, showed that thyroid hormone therapy (for 3–18 months) was associated with reducing the mean thyrotropin value into the normal reference range when compared with placebo (range 0.5–3.7 mIU/L v 4.6–14.7 mIU/L). But no improvement was found in thyroid related symptoms or quality of life.”
Hennessey (2018) [48]	“Persistent symptoms in patients who are biochemically euthyroid with LT4 monotherapy may be caused by several other conditions unrelated to thyroid function, and their cause should be aggressively investigated by the clinician.”
Peterson (2018) [49]	“While the study design does not provide a mechanistic explanation for this observation, future studies should investigate whether preference for DTE is related to triiodothyronine levels or other unidentified causes.”
Bekkering (2019) [50]	“For adults with SCH, thyroid hormones consistently demonstrate no clinically relevant benefits for quality of life or thyroid related symptoms, including depressive symptoms, fatigue
Taylor (2019) [51]	“… many patients have persistent concerns and dissatisfaction with their thyroid hormone replacement.”
de Montmollin 2020 [52]	”In older adults with SCH and high symptom burden at baseline, L-thyroxine did not improve hypothyroid symptoms or tiredness compared with placebo.”
Samuels (2020) [53]	“Serum TSH levels are often measured in patients who report these nonspecific symptoms, and these patients are then treated for mild elevations in TSH that may be unrelated to the presenting symptoms.”
Mitchell (2021) [54]	“The main findings of this survey were a high rate of dissatisfaction with treatment and care. The form of thyroid hormone replacement taken did not correlate with treatment satisfaction.”
Perros (2021) [55]	“Interventions using L- T4 + L- T3 have been disappointing and are unlikely to unlock the persistent symptom enigma for the majority of patients.”
Borson-Chazot (2021) [56]	“In hypothyroidism, QoL appears to be influenced by a number of physiological, behavioral, cognitive and/or lifestyle factors that are not strictly related to thyroid hormone levels.”

## Data Availability

The cited publications of our previous research have appeared as open-source material.
